# NAP1L1 is a prognostic biomarker and contribute to doxorubicin chemotherapy resistance in human hepatocellular carcinoma

**DOI:** 10.1186/s12935-019-0949-0

**Published:** 2019-09-05

**Authors:** Yong Le, Anna Kan, Qi-Jiong Li, Min-Ke He, Hai-Long Chen, Ming Shi

**Affiliations:** 1 Department of Hepatobiliary Oncology, Sun Yat-sen University Cancer Center, Guangzhou, 510060 China; 2 State Key Laboratory of Oncology in South China, Collaborative Innovation Center for Cancer Medicine, Sun Yat-sen University Cancer Center, Guangzhou, China

**Keywords:** NAP1L1, Hepatocellular carcinoma, Prognosis, Chemoresistance

## Abstract

**Background:**

Hepatocellular carcinoma (HCC) is one of the most fatal cancers, and its molecular basis needs to be delineated to identify biomarkers for its potential treatment. The purpose of this study was to identify a novel gene, nucleosome assembly proteins 1-like 1 protein (NAP1L1), associated with aggressive phenotypes of HCC.

**Methods:**

Immunohistochemical staining was used to detect NAP1L1 protein expression in HCC tissues. The prognostic value of NAP1L1 expression was determined using Kaplan–Meier analysis and the Cox proportional hazards model. CCK-8 and apoptosis assays were used to detect the chemosensitivity in vitro. Xenograft tumor models were used to evaluate tumor cell proliferation and chemosensitivity in vivo.

**Results:**

NAP1L1 expression was significantly upregulated in tumor tissues as compared to adjacent non-tumor tissues. High NAP1L1 expression in HCC tissues was associated with aggressive clinicopathologic features, such as serum AFP levels, tumor size and tumor number. Patients with high NAP1L1 expression had poor overall survival in our cohort and in the extra-validation cohort analyzed by TCGA microarray dataset and was further identified as an independent prognostic factor in HCC patients treated with radical resection. Both in vitro and in vivo assays showed that NAP1L1 promoted HCC cell proliferation and contribute to chemotherapy resistance. Further analyses found that some certain stemness associated genes were decreased concurrently with NAP1L1 down-regulation in HCC cell lines.

**Conclusions:**

Our findings support that NAP1L1 is a prognostic biomarker and may contribute to chemotherapy resistance in human hepatocellular carcinoma.

## Background

Hepatocellular carcinoma (HCC) is the fifth most common cancer and the second leading cause of cancer mortality worldwide, and results in more than 700,000 deaths annually [1]. It is particularly prevalent in China, Southeast and Eastern of Asia and sub-Saharan Africa [2]. Most cases of HCC are associated with well-known underlying risk factors, such as chronic viral hepatitis and alcohol abuse. Despite improvements in diagnostic and treatment strategies, the overall survival (OS) of HCC patients remains poor due to postoperative recurrence and metastasis [3, 4]. Therefore, it is urgent to investigate the molecular mechanism involved in HCC initiation and progression, which can be used to help oncologists devise optimal treatment strategies and improve prognoses in HCC patients.

The human Nucleosome assembly proteins 1-like protein (NAP1L) family comprises NAP1L1, NAP1L2, NAP1L3, NAP1L4, NAP1L5, and NAP1L6 [5]. NAP1L1 and L4 expressed ubiquitously in human tissues are highly conserved compared with NAP1L2, L3, and L5, which are expressed predominantly in the brain [6, 7]. The functions of NAP1L proteins that have been attributed include nucleosome assembly, histone transport, histone eviction, transcriptional regulation, and cell cycle progression [8]. NAP1L1 can be detected in most human tissues and cell lines, but increased levels were often found in rapidly proliferating cells [5]. Several studies have identified NAP1L1 being highly expressed in tumors [9–12], which pointed to its potential role in this type of human malignancy. Previous studies also demonstrate that NAP1L1 is over-expressed in fetal liver compared with adult liver [13], in hepatoblastoma compared to healthy adult liver [14]. For now, data regarding the potential role of NAP1L1 in HCC tumorigenesis and progression are limited.Therefore, the frequent aberrant expression of NAP1L1 in HCC tissues was analyzed in our present study. Furthermore, we investigated the prognostic significance of NAP1L1 protein expression levels in HCC patients. Both in vitro and in vivo assays showed that NAP1L1 promoted HCC progression and contribute to chemotherapy resistance.

## Methods

### Patients and samples

This study was approved by the Research Ethics Committee of Sun Yat-Sen University Cancer Center. All patients provided informed consent to participate in the study before they underwent tumor resection. The tissue microarrays consisted of HCC tissues obtained from 304 patients who were diagnosed with HCC between January 2005 and December 2012 at Sun Yat-Sen University Cancer Center. The inclusion criteria were (1) histologically confirmed diagnosis, (2) no neoadjuvant chemotherapy or radiotherapy prior to surgery. Patients with serious complications, other malignant diseases, or no complete follow-up data were excluded from this study. The tumor stage was determined according to the 7th Edition tumor node-metastasis classification system.

### Transarterial chemoembolization treatment after surgery

Transarterial chemoembolization (TACE) was performed using the techniques described in our previous report [15]. Briefly, lobaplatin (50 mg), epirubicin (50 mg), and mitomycin C (6 mg) were mixed in 9 ml of water-soluble contrast medium and 1 ml of sterile water for injection. Depending on the size of, location of, and arterial supply to the tumor, the tip of the catheter was advanced into the segmental artery or specific tumor-feeding artery.

### Immunohistochemical staining

Immunohistochemical (IHC) staining was performed as described in previous study [16]. Briefly, Tissue sections prepared for antigen retrieval by microwave treatment in citrate buffer (pH 6.0) were incubated with anti-NAP1L1 (Sigma, America), anti-Ki67 (Zsbio, China), anti-cleaved-caspase 3 (Affinity, China) primary antibodies. Immunostaining was performed using the Envision System with diaminobenzidine (Dako Cytomation, Glostrup, Denmark). To assess the expression level of NAP1L1 in HCC tissue microarrays, a Vectra-Inform image analysis system (Perkin-Elmer Applied Biosystems) was used as described in previous studies [17, 18].

### Western blot assay

Total protein were extracted using the Protein Extraction Kit (KeyGEN BioTECH, Nanjing, China) according to the manufacturer’s instructions. Protein lysates were separated by 10% SDS-PAGE and then transferred to a PVDF membrane. After the membranes were blocked, they were incubated with various antibodies at 4 °C overnight including, anti-GAPDH (CST, USA), anti-NAP1L1 (Sigma, Germany), anti-NOTCH1 (CST, USA), anti-OCT4 (Santa Cruz, CA, USA), anti-SOX2 (Santa Cruz, CA, USA), anti-c-MYC (CST, USA) and anti-ABCG2 (Abcam, UK). Then, the membranes were incubated with horseradish peroxidase-conjugated antibodies at room temperature for 45 min. Protein signals were detected using enhanced chemiluminescence (Pierce, Rockford, IL, USA).

### RNA extraction, reverse transcription, and real-time PCR

Total RNA was isolated from cell lines using TRIzol Reagent (Invitrogen Life Technologies) according to the manufacturer’s instructions. Each cDNA was synthesized from 2 μg of total RNA using a Revert Aid First–Strand cDNA Synthesis Kit (TOYOBO, Osaka, Japan). For the real-time PCR assay, cDNA was subjected to PCR amplification using SYBR Green (Toyobo, Osaka, Japan) and a Roche LightCycler 480 System. GAPDH was used as an internal control. The primers used in this study were in Additional file 1: Table S1.

### Cell lines and culture conditions

Four human HCC cell lines, i.e., Hep3B, SK-Hep-1, Huh7, and SMMC-7721, and one normal hepatic cell line, i.e., L02, were kindly obtained from the National Cancer Centre Singapore (NCCS). All cell lines were cultured in Dulbecco’s modified Eagle’s medium (DMEM) (Gibco, Carlsbad, CA, USA) supplemented with 10% fetal bovine serum (FBS) (Gibco). The cells were incubated in a humidified incubator supplied with 5% carbon dioxide at 37 °C.

### Plasmid constructs and transfection

The psi-LVRH1GP vector containing short hairpin RNAs (shRNA) targeting NAP1L1 was purchased from GeneCopoeia and transfected into HCC cells using a Lenti-Pac™ HIV Expression Packaging Kit (GeneCopoeia, Inc.) according to the manufacturer’s instructions.

### In vitro cell growth and cytotoxicity assays

The proliferative activity and cytotoxicity assays of cells was determined by CCK8 assay as the manufacture’ instruction (Dojindo, Japan).

### Flow cytometry analysis of apoptotic cells

The SMCC-7721-NC/sh-NAP1L1 cell lines were treated with doxorubicin (DOX, 1 mg/l, KeyGEN BioTECH, Nanjing, China) for 24 h. The sk-hep-1-NC/sh-NAP1L1 cell lines were treated with DOX (2 mg/l) for 24 h. Cell apoptosis was evaluated with the AnnexinV-PI kit (KGA1030, KeyGEN BioTECH, Nanjing, China). Stained cells were analyzed using fluorescence-activated cell sorting (FACS) Canto I or II (BD Bioscience) and FlowJo software.

### Animal study

All the animal experiments were performed in accordance with the guidelines of the Laboratory Animal Ethics Committee of Sun Yat-Sen University. Male BALB/c nude mice (4–5 weeks old) from the Beijing Vital River Laboratory Animal Technology (Beijing, China) were used. Sk-hep-1-sh-NAP1L1 and sk-hep-1-NC cells (1 × 10^6^**/**mice) were subcutaneously inoculated into the right inguinal of the nude mice. After 1 week, the mice were further randomly assigned into the following different groups: Sk-hep-1-sh-NAP1L1 + normal saline (NS), Sk-hep-1-sh-NAP1L1 + DOX, Sk-hep-1-NC + NS, Sk-hep-1-NC + DOX. DOX, Intraperitoneal, 3 mg/kg, twice per week. After treatment for 2 weeks, the subcutaneous tumors were resected, fixed in phosphate-buffered neutral formalin, sectioned serially, and stained with hematoxylin–eosin. Then, immunohistochemical analysis was performed. Tumor volumes was calculated using the formula V = length × width × height/2.

### Statistical analysis

SPSS 20.0 software (IBM, Chicago, IL, USA) and GraphPad Prism V6.0 (GraphPad, La Jolla, CA, USA) were used for statistical analysis. For continuous variables, the data are expressed as the mean ± standard error of the mean. The significance of differences between values was determined using the Student’s *t* test. The Chi squared test was applied to examine the correlation between NAP1L1 expression and clinical pathological parameters. Survival curves for patients were calculated using the Kaplan–Meier method and analyzed using the log-rank test. Prognostic factors were examined by univariate and multivariate analyses using the Cox proportional hazards model. All differences were considered statistically significant with a value of *p *< 0.05.

## Results

### NAP1L1 expression in hepatocellular carcinoma tissues

**Fig. 1:**
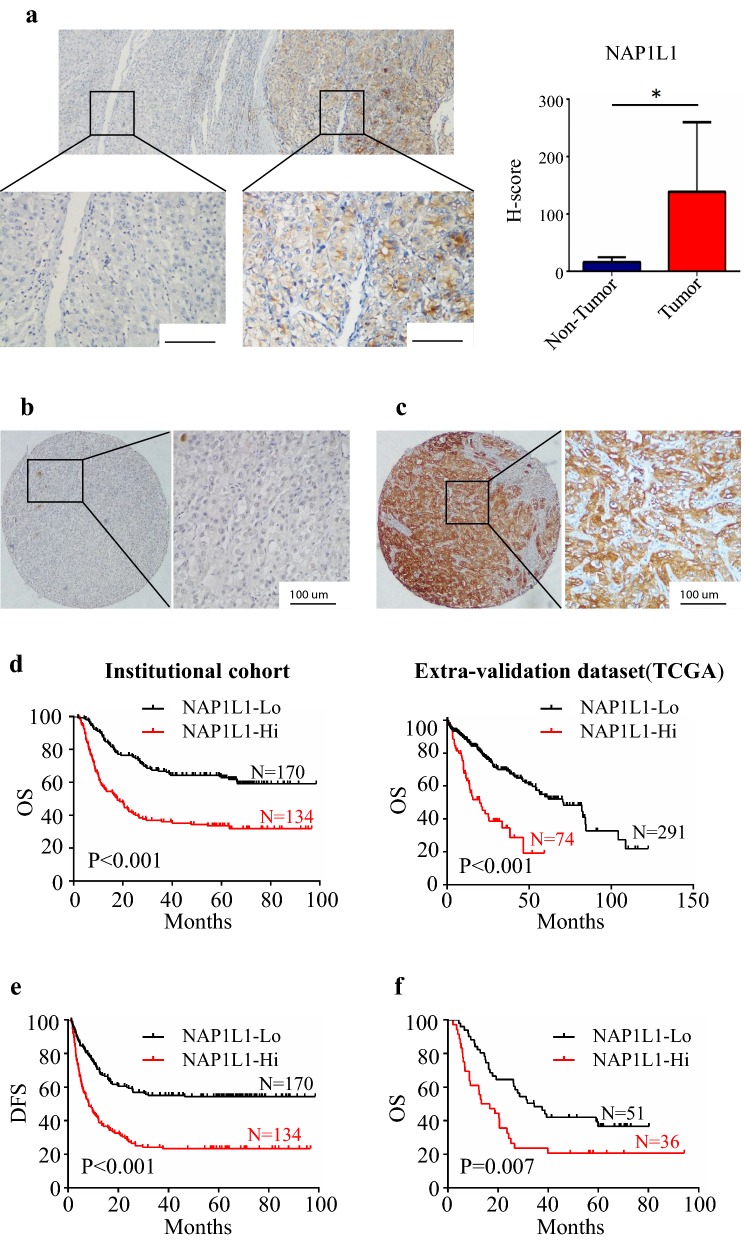
NAP1L1 expression in hepatocellular carcinoma tissues. **a** IHC assays of NAP1L1 expression in adjacent non-tumor tissues and tumor (left ×200, scale bar: 100 μm). NAP1L1 expression levels in tumor tissue are significantly higher than those in adjacent non-tumor tissue (n = 20). The IHC H-scores are shown as mean with SD (right). **b** Representative staining of negative NAP1L1 expression in the tumor cell cytoplasm (left ×40, right ×200; scale bar: 100 μm). **c** Representative staining of positive NAP1L1 expression in the tumor cell cytoplasm (left ×40, right ×200). **d** Kaplan–Meier analysis for OS displayed as the NAP1L1-lo group versus the NAP1L1-Hi group in present study (left). TCGA data (https://www.proteinatlas.org/) further confirmed that high NAP1L1 expression indicated reduced OS of HCC patients (right). **e** Kaplan–Meier analysis for DFS displayed as the NAP1L1-lo group versus the NAP1L1-Hi group in present study. **f** Kaplan–Meier analysis for recurred patients who treated with TACE as the NAP1L1-lo group versus the NAP1L1-Hi group. *IHC* Immunohistochemistry, *SD* standard deviation, *OS* overall survival, *TCGA* The Cancer Genome Atlas, *DFS* disease-free survival, *TACE* transarterial chemoembolization. *p < 0.05

In the preliminary experiment, IHC staining of the HCC specimens showed clear and distinguishable cytoplasm staining for NAP1L1 in tumor tissues, but negative staining in adjacent hepatocytes (Fig. 1a, left). NAP1L1 expression was significantly higher in tumor tissues compared to the adjacent non-tumor tissues (p < 0.05, Fig. 1a, right). To further investigate the correlation between NAP1L1 expression levels and HCC prognosis, we performed IHC staining in specimens from a set of 304 HCC patients. Results showed that the NAP1L1 expression levels in the tumor cell cytoplasm varied widely among different HCC specimens (Fig. 1b, c). Based on NAP1L1 expression in the tumor cell cytoplasm, patients were divided into two groups, the NAP1L1 low group (NAP1L1-Lo; Fig. 1b) and the NAP1L1 high group (NAP1L1-Hi; Fig. 1c).

We next analyzed the relationship between NAP1L1 expression levels in tumor cells and the clinicopathological characteristics. High NAP1L1 expression was significantly associated with aggressive clinicopathologic features (i.e., serum AFP levels, larger tumor size, and late clinical stage) (Table 1). Kaplan–Meier survival analysis revealed that patients in the NAP1L1 high group had worse OS than those in the NAP1L1 low group (p < 0.001) (Fig. 1d, left). Multivariate survival analysis showed that high NAP1L1 expression was an independent prognostic factor for OS in HCC patients after radical resection (hazard ratio = 1.958 95% confidence interval: 1.391–2.755, p < 0.001) (Table 2). Moreover, TCGA data (https://www.proteinatlas.org/) also indicated that patients with high NAP1L1 expression predicted reduced OS of HCC patients (p < 0.001) (Fig. 1d, right) compared to those with low NAP1L1 expression. Meanwhile, the disease-free survival time was significantly lower in the high NAP1L1 group than in the low NAP1L1 group (p < 0.001, Fig. 1e). Multivariate analysis identified that high NAP1L1 expression was also an independent prognostic factor for disease-free survival (Table 2).

**Table 1:** Correlation between NAP1L1 expression and clinicopathologic characteristics (n = 304)

Variable	No.	NAP1L1 expression levels		p value
		Low	High	
Age (years)				
< 50	159	78	81	
≥ 50	145	92	53	*0.012*
Gender				
Female	32	17	15	
Male	272	153	119	0.736
Serum AFP (ng/ml)				
< 400	165	109	56	
≥ 400	139	61	78	*< 0.001*
Cirrhosis				
No	102	61	41	
Yes	202	109	93	0.333
Tumor size (cm)				
< 5	170	115	55	
≥ 5	134	55	79	*< 0.001*
Tumor number				
Solitary	233	138	95	
Multiple	71	32	39	*0.035*
HbsAg				
No	28	22	6	
Yes	276	148	128	*0.011*
Microvascular invasion				
No	200	119	81	
Yes	104	51	53	0.081
BCLC stage				
0–A	228	140	88	
B–C	76	30	46	*0.001*
TNM stage				
I–II	219	133	86	
III–IV	85	37	48	*0.007*

**Table 2:** Univariate and multivariate analysis associated with OS and DFS (n = 304)

Variables	Overall survival			Disease-free survival		
	Univariate p value	Multivariable analysis		Univariate p value	Multivariable analysis	
		HR (95% CI)	p value		HR (95% CI)	p value
Age (years)						
< 50						
≥ 50	0.241			0.246		
Gender						
Female						
Male	0.965			0.352		
HbsAg						
No						
Yes	0.910			0.343		
AFP (ng/ml)						
< 400						
≥ 400	0.079		n.a.	*0.012*		n.a.
Tumor size (cm)						
< 5.0						
≥ 5.0	*< 0.001*	2.548 (1.799–3.607)	*< 0.001*	*< 0.001*	2.417 (1.755–3.328)	*< 0.001*
Tumor number						
Solitary						
Multiple	*< 0.001*	1.532 (1.061–2.212)	*0.023*	*< 0.001*	1.509 (1.054–2.159)	*0.025*
Microvascular invasion						
No						
Yes	*< 0.001*	3.160 (2.227–4.484)	*< 0.001*	*< 0.001*	1.973 (1.415–2.751)	*< 0.001*
NAP1L1 expression						
Low						
High	*< 0.001*	1.958 (1.391–2.755)	*< 0.001*	*< 0.001*	1.876 (1.373–2.565)	*< 0.001*

In our study, 87 patients were recurred after radical resection. The subsequent treatment for those patients were transarterial chemoembolization (TACE). All patients were stratified two groups according to the above criterion. The high NAP1L1 expression group showed dismal OS after TACE treatment than the low NAP1L1 expression group (p = 0.007, Fig. 1f). This results suggested that NAP1L1 expression levels may influence the effects of chemotherapy in HCC.

### Effect of NAP1L1 expression on the prognosis of HCC patients with different TNM stages

**Fig. 2:**
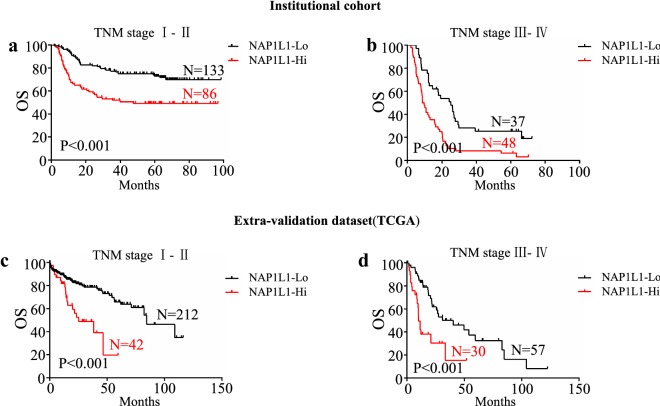
Effect of NAP1L1 expression on the prognoses of patients with different TNM stages. Kaplan–Meier analysis for OS displayed as the NAP1L1 low group versus the NAP1L1 high group in TNM stage I–II (**a**) and stage III–IV (**b**). Kaplan–Meier analysis for OS displayed as the NAP1L1 low group versus the NAP1L1 high group in TNM stage I–II (**c**) and stage III–IV (**d**) based on TCGA database. *OS* overall survival, *TCGA* The Cancer Genome Atlas

We next performed subgroup analysis in the patients with different TNM stages to evaluate predictive value of NAP1L1 protein expression for OS in HCC patients after curative hepatectomy. All patients were stratified according to the TNM seventh staging system. Kaplan–Meier plots of patients with different TNM stages are shown in Fig. 2. Of the 219 patients at stage I–II, 86 patients were identified as having positive NAP1L1 expression in tumor cells. Patients with positive NAP1L1 expression had a poorer surgical prognosis than those with negative NAP1L1 expression in tumor cells (*p *< 0.001, Fig. 2a). Of the 85 patients at stage III–IV, the prognosis of patients with NAP1L1 expression in tumor cells was poorer than that of patients with negative NAP1L1 expression in tumor cells (*p *< 0.001, Fig. 2b). TCGA data (https://www.proteinatlas.org/) confirmed the results that HCC patients with high NAP1L1 expression predicted poorer overall survival in both TNM stage I–II and III–IV, respectively (*p *< 0.001 and *p *< 0.001, Fig. 2c, d).

### NAP1L1 knockdown suppresses tumor growth and increasing chemosensitivity in vitro

**Fig. 3:**
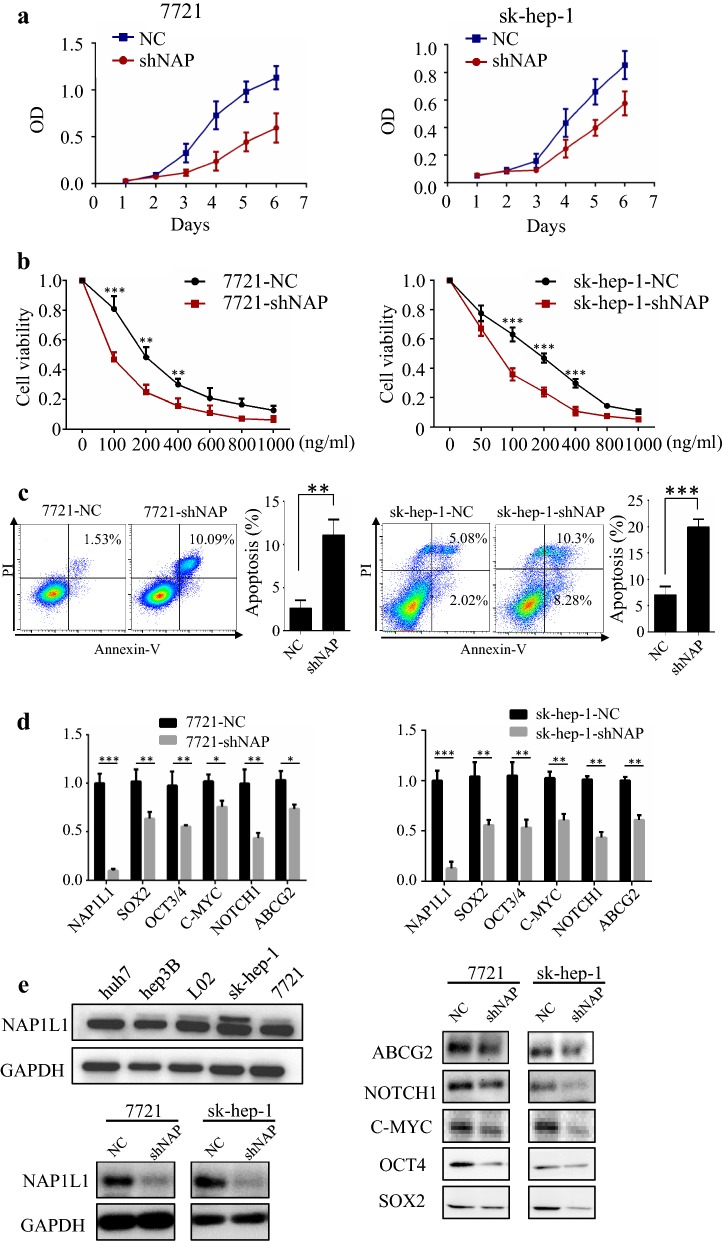
NAP1L1 knockdown suppresses tumor growth and increasing chemosensitivity in vitro. **a** Cell growth of 7721-shNAP1L1, sk-hep-1-shNAP1L1 and the control cells was analyzed by CCK8 assays. **b** Cytotoxicity assays of 7721-shNAP1L1, SK-hep-1-shNAP1L1 and the control cells was analyzed by CCK8 assays. **c** Flow cytometry analysis 7721-shNAP1L1, sk-hep-1-shNAP1L1 and the control cells treated with DOX for 24 h. **d** Cancer stem related markers’ mRNA expression levels were measured in the 7721-shNAP1L1, sk-hep-1-shNAP1L1 and the control cells. **e** Western blotting analyzed NAP1L1 protein levels in different cell lines (left upper). Western blotting showed NAP1L1 significantly downregulated in 7721-shNAP1L1, sk-hep-1-shNAP1L1 compared to their respective control cells (left down). Western blotting analyzed NOTCH1, ABCG2, C-MYC, OCT4 and SOX2 protein level in 7721-shNAP1L1, sk-hep-1-shNAP1L1 and their respective control cells (right). Abbreviations: DOX, doxorubicin; *p *< *0.05,* **p < 0.01, ***p < 0.001

First, we examined the NAP1L1 expression pattern in HCC cell lines (Hep3B, Sk-hep-1, Huh7, SMCC-7721) and normal liver cells (L02). Notably, all cell lines displayed high protein expression levels of NAP1L1 (Fig. 3e, left). To further investigate the role of NAP1L1 in HCC, NAP1L1 was stable knockdown in sk-hep-1 and SMCC-7721 cell lines, respectively (Fig. 3e, right). Scrambled short hairpin RNA (shRNA) was used as a negative control (NC). CCK-8 assay indicated that NAP1L1 knockdown significantly suppressed the proliferation of SMCC-7721 cells (Fig. 3a, left) and sk-hep-1 cells (Fig. 3a, right), respectively. Moreover, cytotoxicity assays and apoptosis assay indicated that downregulated of NAP1L1 increased the sensitivity of SMCC-7721 and Sk-hep-1 cells to doxorubicin (Fig. 3b, c).

Cancer stem cells (CSC) are thought to be responsible for the development, recurrence and chemo-resistance of HCC. Previously studies also demonstrated that NAP1L1 is over-expressed in fetal liver compared with adult liver. In our present study, NAP1L1 was found re-expressed in a subgroup of patients who have HCC and an unfavorable prognosis. Therefore, we sought to evaluate whether the expression of NAP1L1 is correlated with CSC-related markers. We found that some certain CSC-related markers, such as SOX2, OCT3/4, C-MYC, NOTCH1 and ABCG2 were decreased concurrently with NAP1L1 down-regulation in the 7721 cell line (Fig. 3d). Similarly, those CSC-related markers were also decreased in sk-hep-1-shNAP cell line compare to sk-hep-1-NC cell line. Moreover, NOTCH1 and ABCG2 protein expression levels were decreased concurrent with NAP1L1 down-regulation in liver cancer cells by WB analysis (Fig. 3e).

### NAP1L1 knockdown suppresses tumor growth and increasing chemosensitivity in vivo

**Fig. 4:**
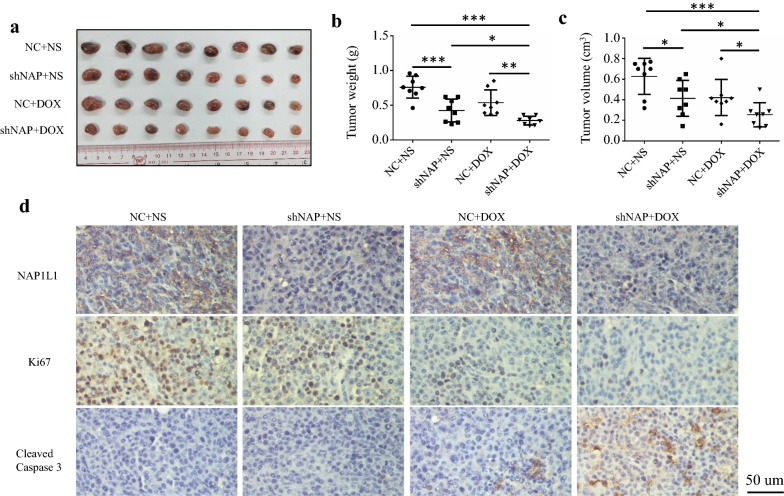
NAP1L1 knockdown suppresses tumor growth and increasing chemosensitivity in vivo. **a** Images of the xenograft tumors formed in nude mice in each group. **b** Tumor weight were summarized in each group. **c** Tumor volume were summarized in each group. **d** Paraffin-embedded tumor sections were stained with anti-NAP1L1, Ki67 or cleaved caspase 3 antibody (scale bar: 50 μm). *DOX* doxorubicin, *NC* negative control, *NS* normal saline; *p < 0.05, **p < 0.01, ***p < 0.001

The stable knockdown cell line, sk-hep-1-shNAP1L1 and the negative control cell line, sk-hep-1-NC were implanted into nude mice to examine the proliferation and chemosensitivity in vivo, respectively. Both tumor weight and tumor volume in sk-hep-1-shNAP1L1 group were significantly lower than those in sk-hep-1-NC group (Fig. 4a–c). Moreover, tumor weight as well as tumor volume in sk-hep-1-sh-NAP1L1 plus DOX group were significantly suppressed compared with that of either agent alone (Fig. 4a–c). IHC analysis in the excised tumor sections demonstrated that Ki67-positive cells were decreased, while cleaved caspase 3-positive cells were increased in the sk-hep-1-sh-NAP1L1 plus DOX group compared with that of either agent alone (Fig. 4d).

## Discussion

Here, we identified the frequent aberrant expression of NAP1L1 in HCC tissues, and this expression pattern was associated with malignant clinicopathological characteristics. Furthermore, multivariate analyses revealed that NAP1L1 expression in tumor cells was an independent and significant risk factor affecting patients’ overall survival and disease-free survival after curative resection.

In previous studies, NAP1L1 expression was reported to be upregulated in several cancers such as renal cancer cell [19], pancreatic neuroendocrine neoplasm [12] and colon cancer [20], but its prognostic value has not yet been reported. Here, we found that NAP1L1 expression was significantly upregulated in HCC as compared to the adjacent non-tumor hepatocytes, and high NAP1L1 expression was significantly associated with aggressive clinicopathologic features (i.e., serum AFP levels, larger tumor size and late clinical stage). Multivariate analysis showed that upregulated NAP1L1 expression was an independent factor for poor prognosis in HCC after curative resection. Furthermore, higher NAP1L1 expression in tumor cells predicted poorer overall survival for TNM stage I-II patients. The findings of our present study suggested that the measurement of NAP1L1 expression in tumor cells could identify worse prognoses among early stage HCC patients. Consistence with our present results, TCGA database suggest that patients with high NAP1L1 mRNA levels had worse OS than those with NAP1L1 low expression.

Cancers have a subpopulation of CSCs or tumor-initiating cells, which have properties shared with normal stem cells [21, 22]. Those cells have aggressive phenotypes in oncogenesis and are resistant to chemotherapies and radiation therapies [23]. Interestingly, studies have demonstrated that NAP1L1 is over-expressed in fetal liver compared with adult liver [13]. In our present study, we demonstrated that NAP1L1 was re-expressed in a subgroup of patients who have HCC and usually indicated an unfavorable prognosis. Moreover, we found that high NAP1L1 expression predict poor survival in patients who treated with TACE. Taken together, we hypothesis that NAP1L1 might be an oncofetal protein in HCC. In vitro and in vivo assays indicated that knockdown NAP1L1 expression in HCC cells increase chemosensitivity. Q-pcr assays also indicated that some certain stemness associated genes, such as SOX2 [24], OCT3/4 [25], NOTCH1 [26], C-MYC [27] and ABCG2 [28] were decreased concurrently with NAP1L1 down-regulation in HCC cells.

The Notch signaling pathway is an evolutionarily conserved pathway and has been reported to promote the self-renewal, proliferation, survival of CSCs in several malignancies [26, 29, 30]. It is one of the most intensively studied candidate therapeutic targets in cancer stem cells, and several Notch inhibitors are being developed [31, 32]. Previous studies has also demonstrated that the Notch1 pathway play important roles in the maintenance and proliferation of liver CSCs [33, 34]. In our present study, data showed that NOTCH1 expression levels were decreased in concurrently with NAP1L1 down-regulation in HCC cell lines by Q-PCR and WB analysis. Meanwhile, the direct target gene of NOTCH1, ABCG2 [35] were also decreased in liver cancer cells which was down-regulated NAP1L1 expression. These above results implied that NAP1L1 down regulation confers liver cancer cells sensitivity to doxorubicin may through inhibiting NOTCH1/ABCG2 signal pathway. However, the specific mechanism that NAP1L1 regulate NOTCH1 expression should be further explored.

## Conclusions

Our study shows that NAP1L1 play an important role in HCC progression and contribute to chemotherapy resistance. The underlying mechanism of NAP1L1 promote HCC progression should be further explored which might enhances the potential of NAP1L1 as a treatment target in HCC.

## Supplementary information

**Additional file 1: Table S1.** Primers for real-time PCR.

## Data Availability

The datasets generated and/or analysed during the current study are available from the corresponding author on reasonable request.

## Abbreviations

HCC: hepatocellular carcinoma
NAP1L1: nucleosome assembly proteins 1-like 1 protein
TCGA: The Cancer Genome Atlas
OS: overall survival
TACE: transarterial chemoembolization
NS: normal saline
DOX: doxorubicin
NC: negative control
IHC: immunohistochemical
CSC: cancer stem cells
Q-PCR: quantitative reverse transcription polymerase chain reaction
WB: western blotting
