# Supplementary material for: NAP1L1 is a prognostic biomarker and contribute to doxorubicin chemotherapy resistance in human hepatocellular carcinoma
Source: Cancer Cell Int. 2019 Sep 5;19:228. doi: 10.1186/s12935-019-0949-0 (PMC6729091; doi:10.1186/s12935-019-0949-0)
Supplement: Supplementary file 1 — Additional file 1: Table S1. Primers for real-time PCR. [file 12935_2019_949_MOESM1_ESM.docx]

Additional file 1: Table S1. Primers for real-time PCR.

| Target gene | Primers |
| --- | --- |
| NAP1L1 | F: TCCTGAAGTTCCTGAGAGTG  R: CACATACATCCTGCTTCACTG |
| C-MYC | F: GTCAAGAGGCGAACACACAAC  R: TTGGACGGACAGGATGTATGC |
| Oct3/4 | F: TGAGGGCGAAGCAGGAGT  R: TCAAAGCGGCAGATGGTC |
| Sox2 | F: TGGGTTCGGTGGTCAAGTC  R: CGCTCTGGTAGTGCTGGGA |
| Notch1 | F: CAACAGCGAGGAAGAGGAGG  R: GCATCAGAGCGTGAGTAGCG |
| GAPDH | F: GCCACATCGCTCAGACAC  R: GCCCAATACGACCAAATCC |
